# Burnout, Depression and Sense of Coherence in Nurses during the Pandemic Crisis

**DOI:** 10.3390/healthcare10010134

**Published:** 2022-01-10

**Authors:** Argyro Pachi, Christos Sikaras, Ioannis Ilias, Aspasia Panagiotou, Sofia Zyga, Maria Tsironi, Spyros Baras, Lydia Aliki Tsitrouli, Athanasios Tselebis

**Affiliations:** 1 Psychiatric Department, “Sotiria” General Hospital of Chest Diseases, 11527 Athens, Greece; irapah67@otenet.gr (A.P.); spyrosbaras@gmail.com (S.B.); lydiatsitrouli@yahoo.gr (L.A.T.); 2 Nursing Department, “Sotiria” General Hospital of Chest Diseases, 11527 Athens, Greece; cris.sikaras@gmail.com; 3 Department of Nursing, University of Peloponnese, 22100 Tripoli, Greece; aspasi@uop.gr (A.P.); zygas@uop.gr (S.Z.); tsironi@uop.gr (M.T.); 4 Department of Endocrinology, “Elena Venizelou” Hospital, 11521 Athens, Greece; iiliasmd@yahoo.com

**Keywords:** depression, sense of coherence, burnout, COVID-19, nurses

## Abstract

During the COVID-19 pandemic, the risk to nurses’ mental health has increased rapidly. The aim of the study was to investigate the prevalence of depression and burnout and to evaluate their possible association with the sense of coherence in nursing staff during the pandemic crisis. The Copenhagen Burnout Inventory questionnaire, Beck’s Depression Inventory, and the Sense of Coherence questionnaire were completed by 101 male and 559 female nurses. Individual and demographic data were recorded. Regarding depression, 25.5% of respondents exhibited mild depression, 13.5% moderate depression and 7.6% severe depression. In the burnout scale, 47.1% had a pathological value. Female nurses had higher burnout (*t* test *p* < 0.01, 49.03 vs. 38.74) and depression (*t* test *p* < 0.01, 11.29 vs. 6.93) scores compared to men and lower levels in the sense of coherence (*p* < 0.05, 59.45 vs. 65.13). Regression evidenced that 43.7% of the variation in the BDI rating was explained by the CBI, while an additional 8.3% was explained by the sense of coherence. Mediation analysis indicated a partial mediation of burnout in the correlation between sense of coherence and depression. The sense of coherence acted as a negative regulator between burnout and depression.

## 1. Introduction

During the COVID-19 pandemic, the risk to nurses’ mental health increased rapidly [1,2,3]. For healthcare workers, the pressure of a professional and social life, along with the occupational hazards associated with exposure to the SARS-CoV-2 virus, lead to increased physical and mental fatigue, as well as to burnout [4,5,6].

Burnout refers to an occupational syndrome associated with emotional and cognitive changes, including emotional exhaustion, depersonalization or cynicism, and diminished feelings of personal effectiveness resulting from chronic work stress [7]. According to Schaufeli and Greenglass, burnout is defined as “a state of physical, emotional and mental exhaustion resulting from long-term involvement in work situations that are emotionally demanding” [8]. Even before the pandemic, nurses had high levels of burnout; studies have shown that burnout can be diagnosed in more than 35% of nurses [9].

The relationship between depression and burnout is a matter of controversy among researchers [10,11]. There is disagreement whether there is an overlap between burnout and depression. More specifically, researchers have argued that since studies have consistently found average to high correlation between depression and burnout, this may indicate overlap, and that burnout may not be a separate psychological phenomenon but a dimension of depression [12]. Kaschka et al. [13] reported that correlations between burnout and depression often occur, indicating that either there is an overlap between burnout and depression, or that burnout is likely to be a risk factor for developing depression. Regarding the similarity of these two entities at the biological level, in their systematic review, Bakusic et al. [14] found that burnout and depression appear to have a common biological basis. On the other hand, researchers [15,16] argue that an important factor that seems to distinguish burnout from depression is the fact that burnout is work-related, while depression is unconfined and pervasive. More specifically, burnout is related to one’s work environment, while depression can occur regardless of environmental conditions (e.g., social or family environment). A recent meta-analysis suggests that although burnout and depression are linked, the magnitude of their relationship is not strong enough to suggest that they are parts of the same construct [17].

Sense of Coherence (SOC) was proposed by Antononsky [18,19] as a construct that expresses the degree to which a person has a diffuse, dynamic but lasting sense that stimuli are internal or external and that stressors are understandable (i.e., predictable, structured and explicable), manageable (i.e., there are resources available to meet the requirements of these stimuli) and meaningful (i.e., the requirements are challenges that are worth committing to and addressing). It has been suggested that a strong SOC helps to manage and deal with stress. This idea is the basis of the “salutogenetic model”; a model that explains how people deal with stressors, such as illness and how people remain reasonably healthy physically and emotionally despite stressors and environmental “insults” [20]. The SOC is often considered to be a stable entity that develops in young adulthood and stabilizes around the age of 30 [20].

From the early 1990s [21] until recently [22], a negative association between depression and SOC is a consistent finding. Studies confirm the effect of SOC on depression in patients with physical illness, such as COPD [23], in multiple sclerosis [24], but also in gynecological cancer [25]. In another study, SOC emerged as a strong predictor of symptoms of adolescent depression [26].

The relationship between SOC and burnout has been much less investigated. A recent study found a negative correlation between them [27], confirming an earlier study [28] with the same results.

There is no study in the literature that examines the role of SOC in the relationship between burnout and depression, although an earlier study [28] simply mentions the existence of correlations between the three variables and concludes that the degree of SOC makes individuals either vulnerable or resistant to both depression and burnout.

The aim of the study was to investigate the prevalence of depression and burnout and to evaluate their possible association with the SOC in nursing staff during the pandemic crisis.

## 2. Subjects and Methods

### 2.1. Research Design

This was a descriptive correlational study. Anonymous self-report questionnaires were used to record the data. To ensure and further protect the anonymity of individuals to whom released data refer, the K-anonymity property was applied [29]. The questionnaires were sent to the emails of nurses who had been randomly selected from lists of Greek professional nurses’ associations. The first page of the electronic questionnaire clearly stated that the completion and submission of the questionnaire was considered a statement of consent. Participation in the research was voluntary. The sample of the study was the nursing staff of Greek public hospitals who responded to the emails. The study was conducted in the second half of March 2021. This study has been approved from the Clinical Research Ethics Committee of “Sotiria” General Hospital (Number 12253/7-5-20) and from the Ethics Committee of the University of Peloponnese (18 January 2021).

### 2.2. Study Participants

With a target population of 27,103 nurses, a confidence level of 99%, a margin of error of 5%, and percentage of our sample picking a particular answer = 50% (we used 50%, which is conservative and provides the largest needed sample size for the given level of accuracy), the minimum sample of the study was set at 651 individuals A total of 850 nurses were invited to answer the questionnaires and 660 agreed to participate in the survey.

### 2.3. Measurement Tools

Demographic and social data from study participants included age, gender, and marital status. Professional information included work experience.

### 2.4. Copenhagen Burnout Inventory

The Copenhagen Burnout Inventory (CBI) is a tool for measuring personal and occupational burnout, consisting of 19 questions. Answers include “always, often, sometimes, rarely, and never/almost never” or “to a very high degree, to a high degree, somewhat, to a low degree and to a very low degree”. The response options are coded in scores of 100, 75, 50, 25, and 0. Possible score range for the burnout scales is 0–100 [29]. Higher scores indicate a higher degree of exhaustion.

The questionnaire includes three subscales:

(I) Personal exhaustion, which assesses the degree of physical and psychological exhaustion the person experiences. It refers to both the physical and psychological exhaustion that accumulates in a person during the day, (e.g.,“How often do you feel physically exhausted?”).
(II) Work-related exhaustion, which assesses the degree of physical and psychological exhaustion the individual perceives about work. It describes work-related exhaustion (e.g.,“Is your job emotionally exhausting?”).
(III) Patient-related exhaustion, which assesses the degree of physical and psychological exhaustion that is considered by the individual to be related to interaction with patients. It depicts exhaustion as a consequence of interpersonal relationships with patients (e.g.,“Does working with patients absorb your energy?”) [30].

For the needs of the study, the Greek adaptation of the questionnaire was used. In reliability analysis, Cronbach’s alpha exceeds 0.7 for all subscales indicating a high level of internal consistency [30]. A score higher than 50 was considered as indicative of burnout [31,32].

### 2.5. Beck’s Depression Inventory

Beck’s Depression Inventory (BDI) measures the cognitive, emotional, behavioral, and physical manifestations of a person’s depression over the past week. It consists of 21 topics which are scored on a scale of 0–3 [33]. The total score is obtained after the sum of the ratings of the 21 topics. The stratification of the severity of depressive symptoms is as follows: 0–9 = without depression, 10–15 = mild depression, 16–23 = moderate depression and ≥24 = severe depression. The scale, in its Greek form [22], is a short and reliable tool for measuring depression and has been used by nursing staff in Greece [34]. Internal consistency and reliability are high and retest reliability ranges between 0.48–0.86 for clinical groups and 0.60–0.90 for non-clinical populations. Validity in relation to an external criterion for depression, such as the clinical diagnosis, is considered to be satisfactory [35].

### 2.6. Sense of Coherence Questionnaire-13

The Sense of Coherence questionnaire (SOC) was developed by Antonovsky to assess how people manage stressful situations and stay well [36]. The SOC-13 scale consists of 13 items, each of which is rated on a Likert scale, ranging from 1 (“very common”) to 7 (“very rare or never”). The scale includes three dimensions: comprehensibility (five items, measuring the person’s perception of the internal and external elements of its environment as structured and predictable); manageability (four items, referring to the person’s ability to meet the demands of stressful environment successfully); and meaningfulness (four items, measuring the person’s ability to view those demands as worthy challenges, thus referring to its motivation) [37,38]. Scores range from 13 to 91, with the highest scores indicating a stronger SOC. In this study we used the short version of SOC-13, which has been standardized in the Greek population and seems to be a reliable and valid instrument, with a Cronbach alpha of 0.83 [37].

### 2.7. Statistical Analysis

**Figure 1:**
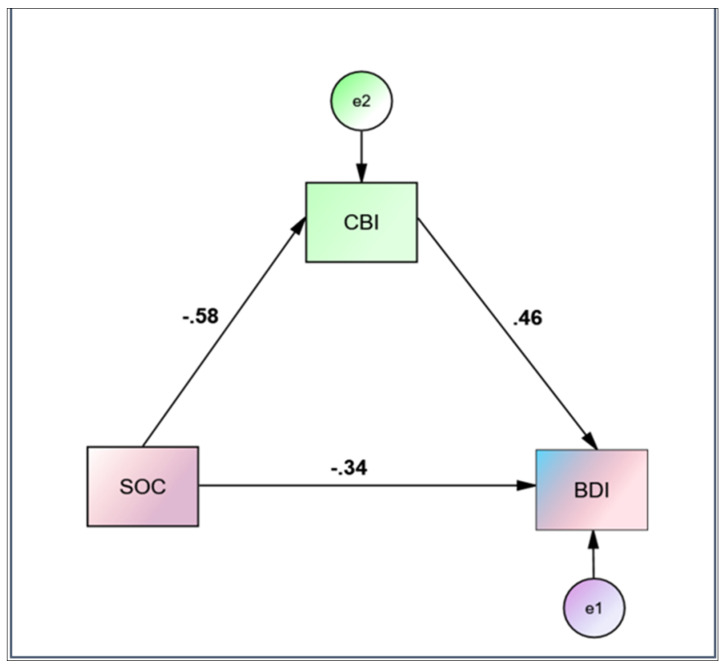
Mediation analysis of Copenhagen Burnout Inventory (CBI) on Sense of Coherence questionnaire (SOC)—Beck’s Depression Inventory (BDI) relationship.

All variables were evaluated using descriptive statistics and values were expressed as means and standard deviations for continuous variables. The prevalence of fatigue and depression was determined as a percentage. Independent *t*-tests were performed to evaluate continuous variables by gender. Analysis of variance (ANOVA) with Bonferroni correction was used to check for differences between groups in continuous variables. Pearson Correlation was performed to determine the strength and direction of the relationship between variables. Linear regression models were built to investigate whether related variables were significant predictors of the independent variable. The evaluation of the linear regression hypotheses (linear relationship, independence, homoscedasticity and normality) was carried out by visual inspection of the variables, residual diagrams and quantile-quantile (QQ) plots. Statistical significance was set at *p* < 0.05 (two-tailed) and analyses were performed using IBM SPSS Statistics 23 (IBM SPSS Statistics for Windows, Version 23.0, IBM Corp, Armonk, NY, USA). Mediation and moderation analyses were conducted using the Hayes SPSS Process Macro. Average scores for the research parameters were compared versus the results obtained in our previous studies in the pre-COVID-19 era [28,35]. IBM SPSS AMOS 23 Graphics was utilized to construct Figure 1.

## 3. Results

A total of 101 men (15.3%) and 559 women (84.7%) nurses participated in the study; 375 nurses reported being married (56.8%), 211 (32.0%) unmarried, and 74 (11.2%) divorced. The sample evidenced no difference in marital status (ANOVA *p* > 0.05). Female nurses had higher burnout values (*t* test *p* < 0.01, 49.03 vs. 38.74) and depression (*t* test *p* < 0.01, 11.29 vs. 6.93), but also lower SOC values compared to men (*t* test *p* < 0.05, 59.45 vs. 65.13, Table 1). Gender differences were statistically significant in both the burnout subscales and the SOC (Table 1).

Regarding depression, 25.5% showed mild depression, 13.5% moderate depression and 7.6% severe depression. On the fatigue scale, 47.1% scored above cutoff. The mean depression score, compared to previous studies [35], was statistically higher in both women (11.29 vs. 8.5 sample *t*-test *p* < 0.01) and in men (6.93 vs. 4.6 sample *t*-test *p* < 0.01). The average SOC was statistically lower compared to previous studies [28] in Greek nurses (60.33 vs. 63.6 sample *t*-test *p* < 0.01).

Significant negative correlations were evidenced among scores on the SOC scale (*p* < 0.01) with both CBI as well as with BDI scales. However, a positive correlation (*p* < 0.01) was indicated between CBI and BDI (Table 2).

A stepwise multiple regression analysis was performed to identify the best predictors for BDI. We defined depression as a dependent variable and as independent variables: work experience, age, burnout and sense of coherence. We tested this model for the absence of multicollinearity. This regression showed that 43.7% of the variation in the BDI score can be explained by the CBI, while an additional 8.3% is explained by SOC; the other variables did not explain the variance in BDI (Table 3).

Bootstrapping was performed with the Hayes SPSS Process Macro to examine whether burnout mediated the relationship between SOC and depressive symptoms: based on 5000 bootstrap samples, a significant indirect relationship between SOC and depressive symptoms was mediated by burnout (Table 4, Figure 1). The outcome variable for the analysis was BDI. The predictor variable for the analysis was SOC. The mediator variable for the analysis was CBI. The indirect effect of CBI on BDI was found to be statistically significant [B= −0.1597, 95% C.I. (−0.1899, −0.1308), *p* < 0.05]. The model explains 42.5% of the variance in the outcome variable. Standardized coefficients for the variables are depicted in Figure 1.

Finally, the moderation role of SOC in the relationship between CBI and BDI was assessed. A simple moderation analysis was performed using the PROCESS method (with CBI as the predictor variable, BDI as the outcome variable and SOC as the moderator variable) (Table 5). The interaction between CBI and SOC was found to be statistically significant [B = −0.0051, 95% CI (−0.0065, −0.0036) *p* < 0.05]. The effect of CBI on BDI showed corresponding results.

**Figure 2:**
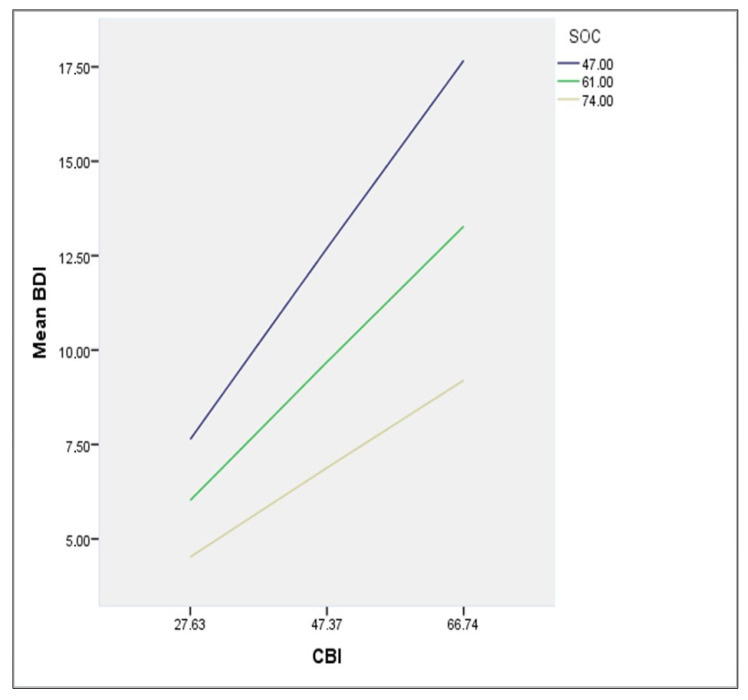
The moderation effect of SOC, between CBI and BDI relationship, at low (47) middle (61) and high (74) degree of SOC.

At low moderation (SOC = 47.00) the conditional effect was 0.2564 [95% CI (0.2230, 0.2898), *p* < 0.05]. At middle moderation (SOC = 61.00) the conditional effect was 0.1855 [95% CI (0.1583, 0.2127), *p* < 0.05]. At high moderation (SOC = 74) the conditional effect was 0.1196 [95% CI (0.0864, 0.1529), *p* < 0.05] (Figure 2). These results identify SOC as a negative moderator of the relationship between CBI and BDI.

## 4. Discussion

A second mental health pandemic is likely to coexist with the COVID pandemic. Nursing staff appear to be particularly vulnerable to the pressure created by the pandemic situation; especially the female population who exhibited higher values of burnout and depression but also lower values of coherence. Scientific studies indicate stronger SOC scores among males compared with females [38,39], but also suggest the dynamic impact of age on SOC [38]. In our sample the female population is significantly younger than males, possibly explaining, in part, the difference in SOC scores, assuming age serves as a confounding variable. Furthermore, research argue that men and women are differentially affected by stressors and make different use of their coping resources [40].These findings are consistent with reviews and meta-analyses indicating that female nurses are more vulnerable to adverse mental health effects; facts that should be taken into consideration in further research on stress, coping, and health [4,41,42].

High rates of burnout and depression in health care workers are a consistent finding in studies worldwide even before COVID-19 [28,43,44]; the pandemic crisis has highlighted the problem. The increase in burnout may be due to the high pressure exerted on hospitals by the pandemic [4]. At the time of the study, cases and admissions to Greek hospitals were on the rise, while work leave for healthcare staff had been suspended for the preceding five months.

In the given period the mental health of the healthcare personnel is especially important for the society as a whole. Firstly, because it has a decisive effect on the quality of health services as mentioned in several studies [45] and, secondly, because it can affect the relationship of public trust in the healthcare system; a factor particularly important in ending the pandemic.

The association between burnout and depression was confirmed in the present study. This correlation (r = 0.66), although being strong, cannot justify an overlap of burnout and depression. The interpretation of the variation of depression from burnout at 43.7% leaves the issue open. Mediation analysis identified burnout as a contributing factor to depression. We would, therefore, prefer to adopt the suggestion given by the World Health Organization, which recognizes burnout as a separate entity, both in International Classification of Diseases (ICD)-10 and ICD-11, giving the code Z 73.0, i.e., as a factor that affects health but not as a separate disease. It is of interest to point out that only one European country has recognized burnout as an occupational disease [46].

The results of the study identify SOC as the negative moderator of the relationship between CBI and BDI. Recent studies highlight the role of SOC in mental health during the pandemic. In particular, a study conducted in eight countries found that a weak SOC was associated with an increased likelihood of “potential depression or anxiety disorder” [47]. A similar result was found in adult samples from Italy [48], and also from Germany [49]. The authors of the latter work explicitly recommended interventions aimed at strengthening the SOC in vulnerable individuals.

Alternatively, interventions have been suggested that use cognitive-behavioral techniques to help people find ways to overcome negative thought patterns and change the way they respond to things that make them feel anxious or upset. Such interventions may include self-help management techniques (e.g., online cognitive behavioral therapy—CBT) [50].

In conclusion, we would like to point out that psychological interventions cannot and should not conceal the real shortcomings of a health system. The theoretical basis of the SOC is to explain why despite the great pressure exerted on them, there were nurses who did not experience burnout and/or depression. The health system is responsible for alleviating the imposed pressure by providing staff with appropriate working conditions, such as adequate supplies and equipment (e.g., PPE), an appropriate patient–nurse ratio, and a work schedule that ensures sufficient rest.

Finally, we will reiterate the message from the World Health Organization for health workers, warning that managing mental health and psychosocial well-being during this pandemic is as important as managing physical health.

The current research was carried out during the COVID-19 pandemic. Therefore, to follow pandemic instructions, data were collected with the online method instead. This meant that nurses without internet access could not participate. Subsequently, the collected data do not represent such groups’ considerations and influences the study’s generalizability. Additionally, the self-reported data were subject to common method biases. Moreover, owing to the periodical rotation of the nursing personnel, contextual factors relating to the unit where the nurses worked were not included in data collection. Finally, the study was cross-sectional. Therefore, causality between the study’s variables cannot be determined.

## 5. Conclusions

We evidenced high rates of depression and burnout in nursing staff. Mediation analysis highlighted burnout as a factor influencing depression, while sense of coherence functioned as a negative moderator between burnout and depression. Psychological, as well as administrative, interventions are necessary to be implemented immediately to address the problem.

## Figures and Tables

**Table 1:** General characteristics of nursing staff and SOC/CBI scores with regards to gender.

P	D. S.	Age	W. E. (in Years)	BDI	Sense of Coherence Questionnaire (SOC)				Copenhagen Burnout Inventory (CBI)			
					Total	A	B	C	Total	Personal Burnout	Work Related Burnout	Patient Related Burnout
Men N = 101	Mean	44.59 *	19.07	6.93 **	65.13 **	24.64 **	18.50 **	21.98 *	38.74 **	38.65 **	42.99 **	33.87 *
	SD	9.42	9.98	5.45	14.01	6.08	5.05	4.76	18.72	18.89	21.77	22.13
Women N= 559	Mean	42.13 *	17.50	11.29 **	59.45 **	21.86 **	16.58 **	20.95 *	49.03 **	52.22 **	54.67 **	39.27 *
	SD	9.89	10.75	8.16	12.94	5.88	4.85	4.39	18.36	19.05	21.67	22.98
Total N = 660	Mean	42.51	17.74	10.62	60.33	22.29	16.88	21.11	47.46	50.14	52.88	38.49
	SD	9.85	10.64	7.95	7.95	5.99	4.93	4.44	18.77	19.63	22.07	22.92

Notes: * independent *t*-test *p* < 0.05; ** independent *t*-test *p* < 0.01. Abbreviations: P, Participants; D.S., Descriptive Statistics; W.E., Work Experience; BDI, Beck’s Depression Inventory; A, Comprehensibility; B, Manageability; C, Meaningfulness.

**Table 2:** Correlations among age, work experience (in years), CBI, SOC, and BDI.

Pearson Correlation N = 660		AGE	Work Experience (in Years)	Sense of Coherence (SOC)	Beck Depression Inventory (BDI)
Work Experience (in Years)	r	0.922 **			
	p	0.001			
Sense of Coherence (SOC)	r	0.114 **	0.075		
	p	0.001	0.054		
Beck Depression Inventory (BDI)	r	0.025	0.038	−0.628 **	
	p	0.518	0.329	0.001	
Copenhagen Burnout Inventory (CBI)	r	0.045	0.094 *	−0.602 **	0.663 **
	p	0.244	0.016	0.001	0.001

Notes: * *p <* 0.05 or ** *p <* 0.01.

**Table 3:** Stepwise multiple regression (only statistically significant variables are included).

Dependent Variable: Beck Depression Inventory (BDI)	R Square	R Square Change	Beta	*t*	*p*
Copenhagen Burnout Inventory (CBI)	0.437	0.437	0.661	22.55	0.01 *
Sense of Coherence (SOC)	0.721	0.083	−0.361	−10.66	0.01 *

Notes: Beta = standardized regression coefficient; correlations are statistically significant at the * *p <* 0.01 level.

**Table 4:** Mediation analysis of Copenhagen Burnout Inventory (CBI) on Sense of Coherence (SOC)–Beck Depression Inventory (BDI) relationship.

Variable	b	SE	*t*	*p*	95% Confidence Interval	
					LLCI	ULCI
SOC → CBI	−0.8515	0.0442	−19.2810	0.001	−0.9382	−0.7648
SOC → BDI	−0.3759	0.0182	−20.6734	0.001	−0.4115	−0.3402
SOC → CBI → BDI	0.1875	0.0143	13.0854	0.001	0.1594	0.2157
Effects						
Direct	−0.2162	0.0203	−10.6601	0.001	−0.2560	−0.1764
Indirect *	−0.1597	0.0149			−0.1899	−0.1308
Total	−0.3759	0.0182	−20.6734	0.001	−0.4115	−0.3402

* Based on 5000 bootstrap samples.

**Table 5:** Moderation analysis: SOC as a negative moderator of the relationship between CBI and BDI.

Outcome Variable: Beck Depression Inventory (BDI)	b	SE	*t*	*p*
Constant	−0.6021 [−6.0401, 4.8359]	2.7694	−0.2174	0.8280
Copenhagen Burnout Inventory (CBI)	0.4945 [0.4037, 0.5853]	0.0463	10.6909	0.001
Sense of coherence (SOC)	0.0246 [−0.0535, 0.1026]	0.0398	0.617	0.5371
Interaction (CBI × SOC)	−0.0051 [−0.0065, −0.0036]	0.0007	−6.9555	0.001

## Data Availability

The data and the questionnaires of the study are available upon request from the corresponding author.
